# From gene modules to gene markers: an integrated AI-human approach selects CD38 to represent plasma cell-associated transcriptional signatures

**DOI:** 10.3389/fmed.2025.1510431

**Published:** 2025-03-12

**Authors:** Basirudeen Syed Ahamed Kabeer, Bishesh Subba, Darawan Rinchai, Mohammed Toufiq, Taushif Khan, Marina Yurieva, Damien Chaussabel

**Affiliations:** ^1^Department of Pathology, Saveetha Medical College and Hospital, Saveetha Institute of Medical and Technical Sciences (SIMATS), Saveetha University, Chennai, India; ^2^The Jackson Laboratory for Genomic Medicine, Farmington, CT, United States; ^3^St Jude Children’s Research Hospital, Memphis, TN, United States

**Keywords:** large language models, gene prioritization, targeted transcriptional profiling, plasma cell responses, CD38, biomarker discovery

## Abstract

**Background:**

Knowledge-driven prioritization of candidate genes derived from large-scale molecular profiling data for targeted transcriptional profiling assays is challenging due to the vast amount of biomedical literature that needs to be harnessed. We present a workflow leveraging Large Language Models (LLMs) to prioritize candidate genes within module M12.15, a plasma cell-associated module from the BloodGen3 repertoire, by integrating knowledge-driven prioritization with data-driven analysis of transcriptome profiles.

**Methods:**

The workflow involves a two-step process: (1) high-throughput screening using LLMs to score and rank the 17 genes of module M12.15 based on six predefined criteria, and (2) prioritization employing high-resolution scoring and fact-checking, with human experts validating and refining AI-generated scores.

**Results:**

The first step identified five candidate genes (CD38, TNFRSF17, IGJ, TOP2A, and TYMS). Following human-augmented LLM scoring and fact checking, as part of the second step, CD38 and TNFRSF17 emerged as the top candidates. Next, transcriptome profiling data from three datasets was incorporated in the workflow to assess expression levels and correlations with the module average across various conditions and cell types. It is on this basis that CD38 was prioritized as the top candidate, with TNFRSF17 and IGJ identified as promising alternatives.

**Conclusion:**

This study introduces a systematic framework that integrates LLMs with human expertise for gene prioritization. Our analysis identified CD38, TNFRSF17, and IGJ as the top candidates within the plasma cell-associated module M12.15 from the BloodGen3 repertoire, with their relative rankings varying systematically based on specific evaluation criteria, from plasma cell biology to therapeutic relevance. This criterion-dependent ranking demonstrates the ability of the framework to perform nuanced, multi-faceted evaluations. By combining knowledge-driven analysis with data-driven metrics, our approach provides a balanced and comprehensive method for biomarker selection. The methodology established here offers a reproducible and scalable approach that can be applied across diverse biological contexts and extended to analyze large module repertoires.

## 1 Introduction

The development of targeted transcriptional profiling assays is crucial for translating large-scale molecular profiling data into actionable clinical insights (1–4). These assays enable precise, quantitative assessments of the abundance of panels comprising tens to hundreds of transcripts, offering advantages such as cost-effectiveness, rapid turnaround times, and the ability to process large sample numbers (5–7). However, the critical task of selecting relevant candidate genes for inclusion in targeted assays can be challenging, especially when contending with the extensive volumes of biomedical information generated by systems-scale profiling technologies (8).

Knowledge-driven methods for candidate gene prioritization must efficiently sift through vast amounts of literature to identify the most promising candidates. This process can be lengthy and may lack depth due to the sheer volume of information available for each gene. While resources such as gene ontologies and curated pathways can help, they often provide only superficial information about the genes and may lack context (9).

Given these limitations, there is a clear need for more efficient and comprehensive methods to prioritize candidate genes from large-scale molecular profiling data. The introduction of Large Language Models (LLMs) has opened up new possibilities for leveraging collective biomedical knowledge in candidate gene prioritization. LLMs, such as GPT-4 (OpenAI), Claude (Anthropic), and PaLM (Google), have demonstrated remarkable capabilities in natural language understanding and generation (10–12). Building upon previous work demonstrating the utility of LLMs in manual candidate gene prioritization (13), we sought to further streamline the process by developing an automated LLM-based workflow. This automated approach aims to enable the prioritization of extensive module repertoires, such as BloodGen3, and facilitate the design of disease-specific panels.

In the current study, we focus on module M12.15, a plasma cell-associated module from the BloodGen3 repertoire. The plasma cell signature captured by module M12.15 has been linked to various physiological and pathological conditions, including antibody responses to vaccines, autoimmune diseases, and certain hematological malignancies (14–17). Our stepwise approach leverages the capabilities of LLMs to score and rank candidate genes based on predefined criteria and incorporate reference transcriptome data to guide the final selection. We introduce a novel human-in-the-loop augmented scoring process, where human experts validate and refine the LLM-generated scores, ensuring accuracy and relevance. Through this process, we ultimately identify a top candidate gene for module M12.15, showcasing the potential of LLMs to enhance the efficiency and scalability of knowledge-driven candidate biomarker prioritization.

## 2 Methods

### 2.1 BloodGen3 module repertoire

This study employs the BloodGen3 module repertoire, a comprehensive framework for blood transcriptome analysis developed by Altman et al. (14). The repertoire was constructed using 16 reference whole blood transcriptome datasets, encompassing 985 distinct transcriptional profiles across 16 medical conditions: B-cell deficiency, chronic obstructive pulmonary disease (COPD), pregnancy, multiple sclerosis (MS), juvenile dermatomyositis (JDM), post-liver transplantation (liver transplant), melanoma, human immunodeficiency virus infection (HIV), tuberculosis (TB), sepsis, *Staphylococcus aureus* infection (Staph), systemic lupus erythematosus (SLE), influenza virus infection (influenza), respiratory syncytial virus infection (RSV), Kawasaki disease (Kawasaki), and systemic onset juvenile idiopathic arthritis (SoJIA). Through co-expression analysis, 382 modules were identified, each representing a set of genes exhibiting coordinated expression patterns across diverse pathological conditions. These modules are further organized into higher-level structures termed aggregates, where each aggregate comprises multiple modules sharing similar expression characteristics across the reference cohorts.

### 2.2 Large language models

To facilitate the prioritization and selection of candidate genes, we utilized state-of-the-art LLMs. Specifically, we employed GPT-4 (developed by OpenAI), Claude 3 (created by Anthropic), and Consensus GPT (a specialized AI research assistant integrated with ChatGPT) (18–20). GPT-4 is an advanced autoregressive language model with over 1 trillion parameters, capable of generating human-like text by leveraging patterns learned from exposure to a vast corpus of internet data (19) Claude 3, on the other hand, incorporates constitutional AI techniques alongside its extensive parameter count, ensuring outputs align with predefined constraints (18).

Consensus GPT, built on the foundation of GPT-4, has access to over 200 million academic papers, providing a more comprehensive and potentially more accurate evaluation compared to generic LLMs. It is specifically designed for scientific literature analysis and fact-checking (20).

These models represent significant advancements in natural language processing and generation, offering improved performance and reliability compared to their predecessors. By employing multiple LLMs, we aimed to leverage the strengths of each model and enhance the robustness of our gene prioritization process.

### 2.3 Module selection for candidate gene prioritization (step 1)

The initial step in our workflow involves selecting a module from the BloodGen3 repertoire for candidate gene prioritization. This selection is guided by several considerations, including: (1) association with specific cell types or biological processes, as determined by prior research (14–17); (2) abundance pattern across reference patient cohorts, which can provide insights into its potential clinical relevance; and (3) connection to various disease states and physiological conditions, as established by previous studies and published literature. For the current study, we focused on module M12.15, which our prior work has linked to plasma cell activity and antibody production (14).

### 2.4 LLM-driven scoring of module genes (step 2)

To enhance the robustness of our gene prioritization process, we employed two distinct LLM scoring approaches that reflect the advancements in LLM capabilities over the years since the initiation of this research.

#### 2.4.1 Step 2a: LLM chat scoring

Following the scoring approach described by Toufiq et al. (13), we utilized OpenAI’s GPT-4 and Anthropic’s Claude to score the genes within the selected module. Each LLM was tasked with scoring the genes on a scale of 0 to 10 based on six criteria, providing an evaluative comment and supporting references when applicable. The criteria included:

- Association with plasma cell responses: Scored based on evidence of the gene’s role in modulating or responding to plasma cell-related processes, including B cell differentiation, activation, antibody secretion, immunoglobulin production, or involvement in signaling pathways pertinent to plasma cell functions.
- Relevance to circulating leukocytes immune biology: Scored based on evidence linking the gene to the development, function, or regulation of circulating leukocytes, including impacts on leukocyte differentiation, activation, signaling, or effector functions;
- Current use as a biomarker in clinical settings: Scored based on evidence of the gene or its products’ application as biomarkers for diagnosis, prognosis, or monitoring of diseases in clinical settings, with a focus on their validated use and acceptance in medical practice.
- Potential value as a blood transcriptional biomarker: Scored based on evidence supporting the gene’s expression patterns in blood cells as reflective of specific physiological or pathological states, considering both current research findings and potential for future clinical utility;
- (e) known drug target status: Scored based on evidence of the gene or its encoded protein serving as a target for therapeutic intervention, including approved drugs targeting this gene, compounds in clinical trials, or promising preclinical studies;
- (f) therapeutic relevance for diseases involving the immune system: Scored based on evidence linking the gene to the pathogenesis, progression, or treatment of diseases involving the immune system, including its role in immune dysregulation, or as a target for immunotherapy.

The scoring criteria ranged from 0 (no evidence found) to 10 (strong evidence), with intermediate scores reflecting varying levels of evidence and validation. The model’s output was structured as a table, with genes as rows and columns for gene names and scores for each criterion (a–f). This systematic scoring approach allowed for a comprehensive evaluation of each gene’s relevance to plasma cell biology, immune function, and potential clinical applications.

#### 2.4.2 Step 2b: LLM high-throughput chat scoring

To leverage the enhanced capabilities of LLMs and to efficiently process larger gene sets, we employed Claude 3.5 Sonnet for a high-throughput scoring approach. This method allowed for the evaluation of genes in larger batches (up to 10 genes), potentially reducing bias and increasing efficiency. The scoring was run in triplicates, and the scores were averaged to enhance reliability and account for potential variations in LLM outputs.

### 2.5 Selection of top candidates (step 3)

We first ranked the genes based on the cumulative scores generated by the LLMs and then identified the top five candidates selected by each LLM. These candidates were pooled and subjected to further analysis in the next step.

### 2.6 High-resolution scoring and human-in-the-loop fact-checking using consensus GPT (step 4)

Following the initial scoring by GPT-4 and Claude 3.5, we implemented a more rigorous, human-augmented scoring and fact-checking process using the Consensus GPT app, a custom GPT model available in the OpenAI Plus environment.^1^ This step was designed to provide a more detailed and evidence-based evaluation of the top-scoring genes identified in Step 2. We prompted Consensus GPT to generate scores for each of the six criteria, along with justifications and references. This process was repeated for each of the top-scoring genes identified in Step 2. Crucially, a human expert then evaluated the backing references provided by Consensus GPT for accuracy and relevance. When discrepancies or inadequacies were identified in the AI-generated content, the human expert prompted Consensus GPT to revise its evaluation, providing additional context or pointing to more appropriate references as necessary. This iterative, human-in-the-loop approach ensured that the final scores and justifications were not only comprehensive but also verified by human expertise. The process allowed for a more nuanced and accurate evaluation of the scientific literature supporting each gene’s relevance to plasma cell biology, immune function, and potential clinical applications.

### 2.7 Refinement of candidate gene selection (step 5)

In this step, we further refined the selection of the top candidate gene by incorporating additional transcriptome profiling data from three different datasets previously deposited by us: a reference RNA-seq dataset (GSE60424) (21), and a dataset from the Molecular Signature in Pregnancy (MSP) study (PRJNA898879) (4), which comprises 88 samples collected at 6 of ∼15 available time points from 15 women with uncomplicated pregnancies, as well as a comprehensive microarray dataset covering 16 disease states and physiological conditions (GSE100150) (14). Refinement of candidate gene selection involved two sub-steps: (1) flagging the candidate with low expression, and (2) flagging the candidate with low correlation to the module average.

#### 2.7.1 Step 5.1: flagging candidates with low expression

The expression data was provided to GPT-4 in a CSV file format. GPT-4 was instructed to apply a combined filter to identify genes suitable for reliable measurement in an RT-PCR assay based on their expression levels. The filtering criteria were: (1) a median count of at least 50 across all samples; (2) expression levels greater than 15 in at least 50% of the samples. GPT-4 calculated these metrics for each gene and generated a summary table including: (1) the median count of each gene across all samples; (2) the percentage of samples where each gene is expressed at levels greater than 15; (3) a flag indicating whether each gene meets both criteria. Genes that did not meet both criteria were flagged as potentially challenging to measure reliably in the targeted assay.

#### 2.7.2 Step 5.2: flagging candidates with low correlation to module average

We compiled a CSV file for each dataset containing correlation coefficients between the expression levels of individual genes within the M12.15 module and the average expression of all genes in that module. GPT-4 was instructed to analyze this data and filter out genes that are not representative of the module’s behavior across these conditions. To filter these gene candidates, we used the following criteria: (1) median correlation coefficient across all reference cohorts for each gene; (2) percentage of reference cohorts in which each gene’s correlation coefficient exceeds our cut-off; (3) identification of genes with exceptionally low correlation coefficients that fall below the lower bound of the Interquartile Range (IQR). GPT-4 generated a comprehensive table including gene symbol or identifier, median correlation coefficient across all cohorts, percentage of cohorts in which the gene’s correlation coefficient is above our cut-off, and a Boolean indicator showing whether the gene is considered an outlier based on exceptionally low correlations. The table was sorted by the Median Correlation in descending order to highlight the most representative genes at the top. Genes that did not meet both criteria were flagged as potentially less reliable surrogates for the module.

### 2.8 Utilization of LLMs for manuscript preparation

In addition to the development and application of the automated gene prioritization workflow, we also explored the potential of LLMs in assisting with the preparation of this manuscript. Specifically, Claude 3.5, developed by Anthropic, was utilized for this task. The paper by Toufiq et al. (13) and a manuscript we wrote focusing on the prioritization of M14.51, which used the same methodology and workflow, were loaded as context, providing background information and a foundation for Claude to build upon. Data, figures, and key findings from the current study were also provided to the LLM. Claude was employed in an iterative process to generate text from outlines and following general instructions. This process involved multiple rounds of revisions at different levels (section, paragraph) as needed. The AI assistant was also used for editing and refining the content to ensure clarity, coherence, and adherence to scientific writing conventions. All AI-generated text was reviewed and validated by the human authors, who provided additional context, corrections, and interpretations as needed.

## 3 Results

### 3.1 Selection and prioritization of module M12.15

**Figure 1:**
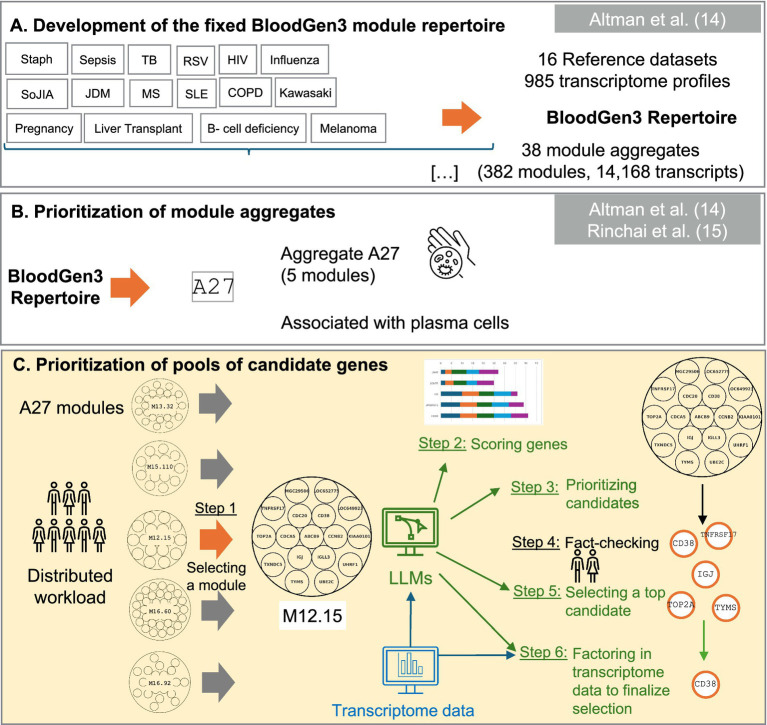
Schematic overview of the targeted panel development strategy. This figure presents our novel workflow for candidate gene prioritization **(Panel C)** within a broader omics data-driven strategy for developing targeted transcriptome fingerprinting assays (TFAs). **(Panel A)** illustrates the data-driven construction of co-expressed blood transcriptional modules derived from 16 reference datasets, encompassing 985 transcriptome profiles. This “fixed transcriptional repertoire” comprises 382 modules organized into 38 aggregates, representing 14,168 transcripts analyzed across patients with sixteen distinct medical conditions: B-cell deficiency, chronic obstructive pulmonary disease (COPD), pregnancy, multiple sclerosis (MS), juvenile dermatomyositis (JDM), post-liver transplantation (liver transplant), melanoma, human immunodeficiency virus infection (HIV), tuberculosis (TB), sepsis, *staphylococcus aureus* infection (Staph), systemic lupus erythematosus (SLE), influenza virus infection (influenza), respiratory syncytial virus infection (RSV), Kawasaki disease (Kawasaki), and systemic onset juvenile idiopathic arthritis (SoJIA). **(Panel B)** demonstrates how the application of BloodGen3 across multiple studies provided insights into the biological and clinical relevance of its modular signatures, leading to the identification of module aggregate A27, which shows strong associations with plasma cells, vaccine responses, and B-cell disorders. This module was subsequently prioritized for inclusion in a generic Immune Profiling TFA panel (ImmP-TFA). **(Panel C)** illustrates our novel workflow that leverages Large Language Models (LLMs) for prioritizing candidate genes, providing a systematic approach for comprehensive characterization and evaluation of candidates for potential inclusion in the ImmP-TFA panel.

**Figure 2:**
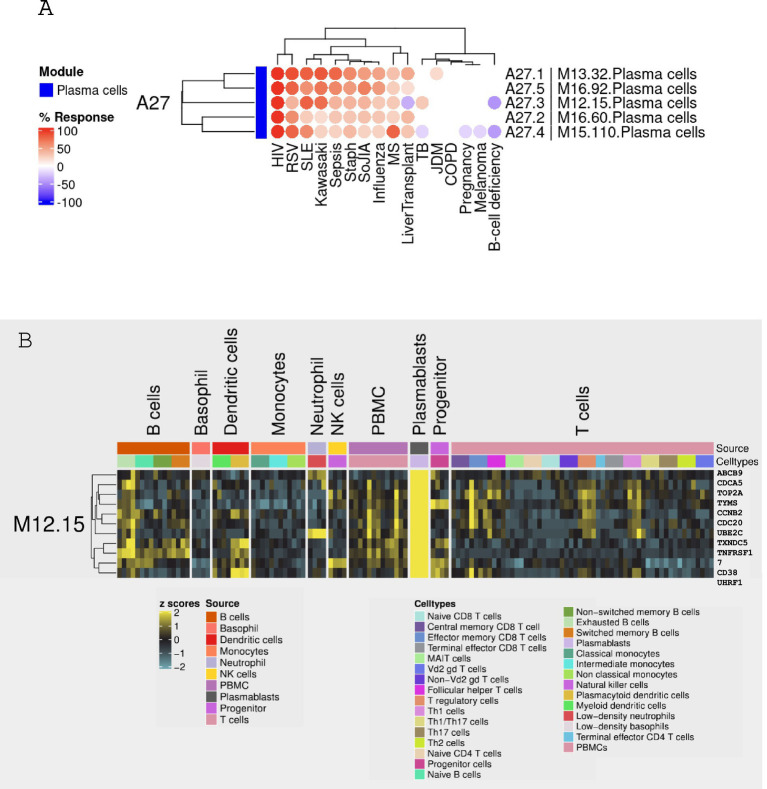
Expression patterns of module M12.15 across 16 reference patient populations. **(A)** Heatmap showing the relative abundance of module M12.15 across 16 distinct medical conditions: patients with B-cell deficiency, chronic obstructive pulmonary disease (COPD), pregnancy, multiple sclerosis (MS), juvenile dermatomyositis (JDM), post-liver transplantation (liver transplant), melanoma, human immunodeficiency virus infection (HIV), tuberculosis (TB), sepsis, *Staphylococcus aureus* infection (Staph), systemic lupus erythematosus (SLE), influenza virus infection (influenza), respiratory syncytial virus infection (RSV), Kawasaki disease (Kawasaki), and systemic onset juvenile idiopathic arthritis (SoJIA). The color scale represents standardized expression levels (red: higher expression; blue: lower expression). **(B)** Heatmap visualizing module M12.15 expression across distinct cell types and subsets. Cell populations include plasmablasts, B cells, basophils, dendritic cells, monocytes, neutrophils, Natural Killer (NK) cells, Peripheral Blood Mononuclear Cells (PBMC), progenitor T cells, and various T cell subsets. Yellow indicates higher expression, while blue indicates lower expression. Data sourced from Monaco et al. (25).

The current study focused on module M12.15, a component of the BloodGen3 module aggregate A27. Detailed information pertaining to the module construction is illustrated in Figure 1. This module was selected for further analysis based on its expression patterns observed across a sample of 16 reference patient populations (Figure 2A). Moreover, the presence of genes such as CD38, IGJ (Immunoglobulin J chain), and TNFRSF17 (also referred to as BCMA, B-cell maturation antigen) in module M12.15 suggests a potential association with plasma cell responses and antibody synthesis, given their well-established roles in the biology of plasma cells (22–24). This association is further supported by the module’s higher expression in plasmablasts and B cells compared to other cell types, as evident from the heatmap depicting the module’s expression across different cell subsets (Figure 2B) (25).

### 3.2 Dual LLM scoring approach employing chat-GPT-4 and Claude for M12.15 gene prioritization

Module M12.15 encompasses 17 genes: ABCB9, CCNB2, CD38, CDC20, CDCA5, IGJ, IGLL3, KIAA0101, LOC649923, LOC652775, MGC29506, TNFRSF17, TOP2A, TXNDC5, TYMS, UBE2C, and UHRF1. To prioritize these genes, we implemented two distinct scoring methodologies leveraging the capabilities of LLMs: an initial methodology (Step 2a) employing GPT-4 and Claude, and an alternative approach (Step 2b) utilizing Claude 3.5 in high-throughput mode (see methods for details).

**Figure 3:**
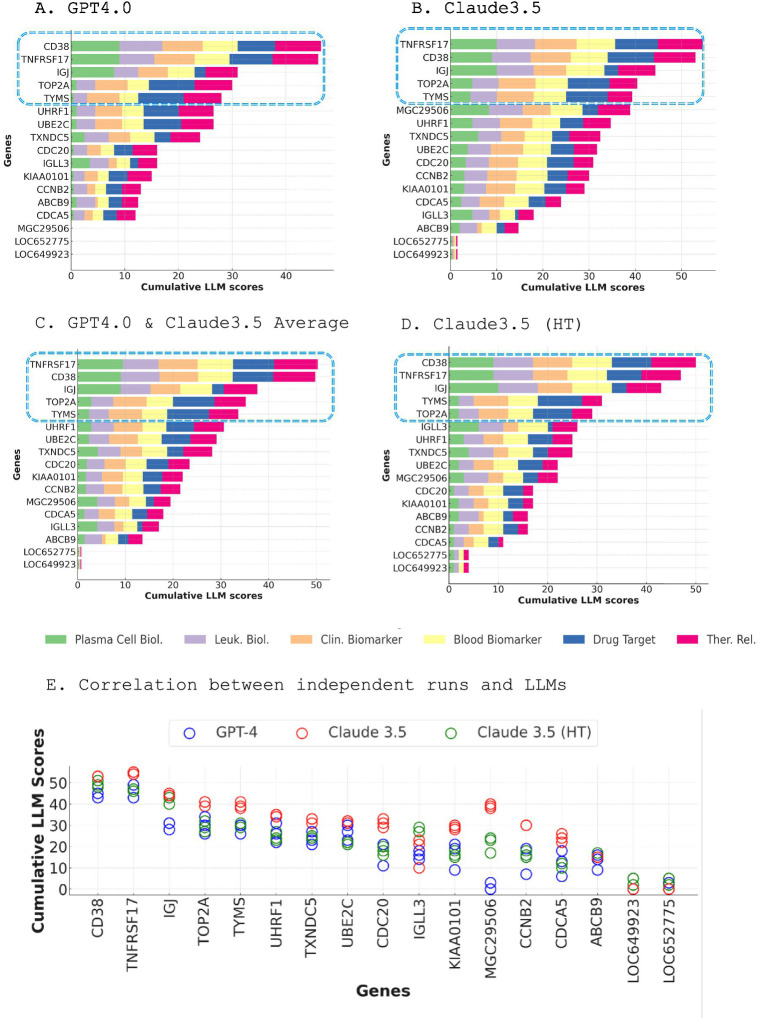
Dual LLM scoring approach for prioritizing genes within module M12.15. **(A)** Cumulative scores of M12.15 genes generated by GPT-4, with CD38 emerging as the top candidate **(A)**. Cumulative scores of M12.15 genes generated by Claude 3.5, with TNFRSF17 ranking as the top candidate (**B**). Average scores from GPT-4 and Claude 3.5, revealing TNFRSF17 as the top candidate, followed by CD38 (**C**). Claude 3.5 high-throughput scoring results, consistent with the averaged scores, with TNFRSF17 and CD38 maintaining their positions as the leading candidates (**D**). Correlation matrix illustrating the consistency of scoring results across three independent runs for each LLM and three distinct LLMs (**E**).

Figure 3 illustrates the results from both scoring methods. Remarkably, both approaches identified the same set of genes as the top five candidates: CD38, TNFRSF17, IGJ, TOP2A, and TYMS. In the GPT-4 scoring, CD38 emerged as the leading candidate with the highest cumulative score, closely followed by TNFRSF17 (Figure 3A). IGJ, TOP2A, and TYMS also received notable scores. In the Claude 3.5 scoring, TNFRSF17 emerged as the top candidate with the highest cumulative score, with CD38 ranking second (Figure 3B). Averaging the scores from GPT-4 and Claude 3.5 revealed TNFRSF17 as the top candidate, followed by CD38 (Figure 3C). The Claude 3.5 high-throughput scoring is consistent with these findings, with TNFRSF17 and CD38 maintaining their positions as the leading candidates, and IGJ, TOP2A, and TYMS securing the third, fourth, and fifth places, respectively (Figure 3D).

Intriguingly, while CD38 and TNFRSF17 consistently scored highly across all selection criteria, the other top genes exhibited more variable profiles. IGJ obtained a high score for plasma cell biology but lower scores for drug target potential, suitability as a blood biomarker, and clinical relevance. Conversely, TOP2A (26) and TYMS received lower scores for plasma cell and leukocyte biology but ranked highly as potential drug targets, blood biomarkers, clinical markers, and therapeutic targets (27–32).

The robustness and consistency of the scoring results were underscored by the excellent correlation observed between the three independent runs for each LLM and across the three distinct LLMs (Figure 3E). This strong agreement across diverse models and iterations lends further credence to the selection of top-tier candidate genes.

### 3.3 High-resolution scoring and fact-checking prioritizes top M12.15 candidates

We employed Consensus GPT to further refine our top candidate selection. Consensus GPT scored each gene across six criteria, providing justifications and references for scores of 4 or above. Human experts then verified these references and critically evaluated the scores. When necessary, experts prompted the model to reassess its evaluations, offering additional context or highlighting overlooked literature. This iterative, human-in-the-loop process allowed for refinement of the AI-generated assessments. This process, although more labor-intensive, provides a level of scrutiny and validation essential for confident gene selection.

**Figure 4:**
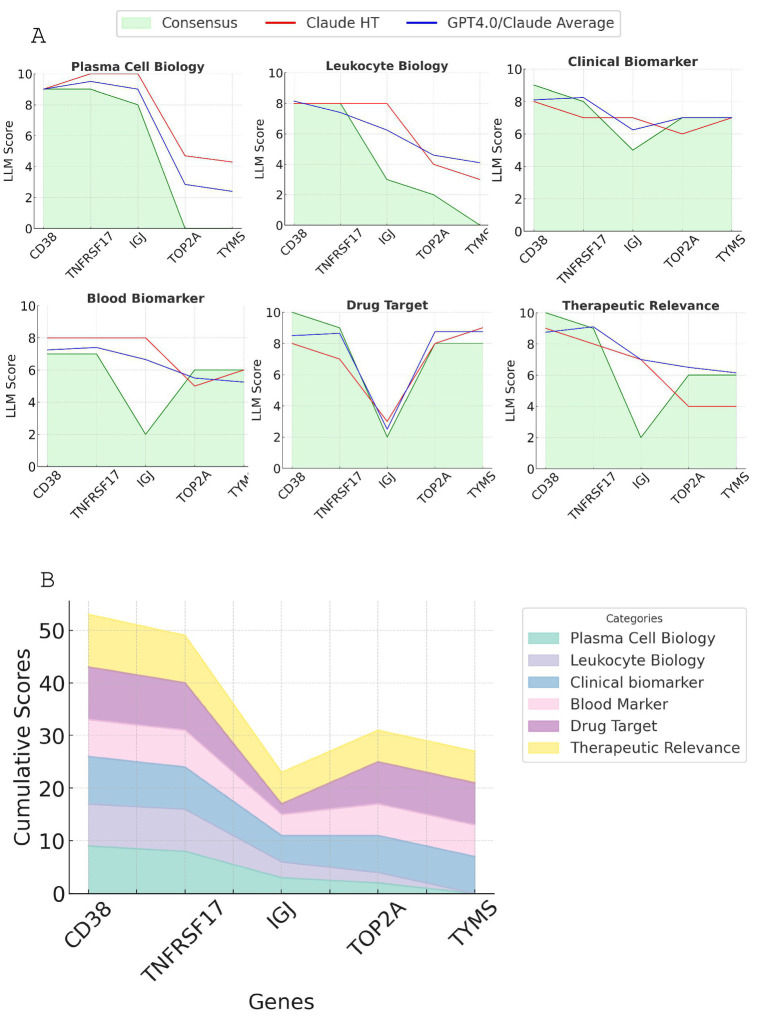
Comparison of scoring results from LLM approaches for the top candidate genes. **(A)** The line plot displaying the scores generated by Claude3.5/GPT, Claude3.5 high-throughput, and Consensus GPT across six key criteria: plasma cell biology, leukocyte biology, clinical biomarker potential, drug target status, blood biomarker potential, and therapeutic relevance. **(B)** Stacked line graph showing the consensus GPT scoring for all six criteria for the top five candidate genes. The cumulative scores are represented by the height of each stacked line.

Figure 4 compares the scoring results from three different LLM approaches - Claude3.5/GPT, Claude3.5 high-throughput, and Consensus - across six key criteria. The scoring patterns appear to be relatively consistent across the three LLM approaches for each criterion, with some minor variations. CD38 and TNFRSF17 consistently emerged as the top two candidates across all approaches. With Consensus GPT, CD38 generally generated a higher or equal score compared to TNFRSF17, but both demonstrated strong performance across multiple criteria. The Consensus scoring, being the most comprehensive and rigorous approach, serves as the basis for the final gene prioritization.

A detailed breakdown of scores and justifications for each gene across the six criteria, complete with supporting references and evaluative comments, is provided in Supplementary Table 1. Based on this comprehensive analysis, we conclude that CD38 and TNFRSF17 are the most promising candidate genes for module M12.15, exhibiting strong performance across multiple key criteria.

### 3.4 Refinement of gene selection for module M12.15

#### 3.4.1 Flagging candidates with low expression

**Figure 5:**
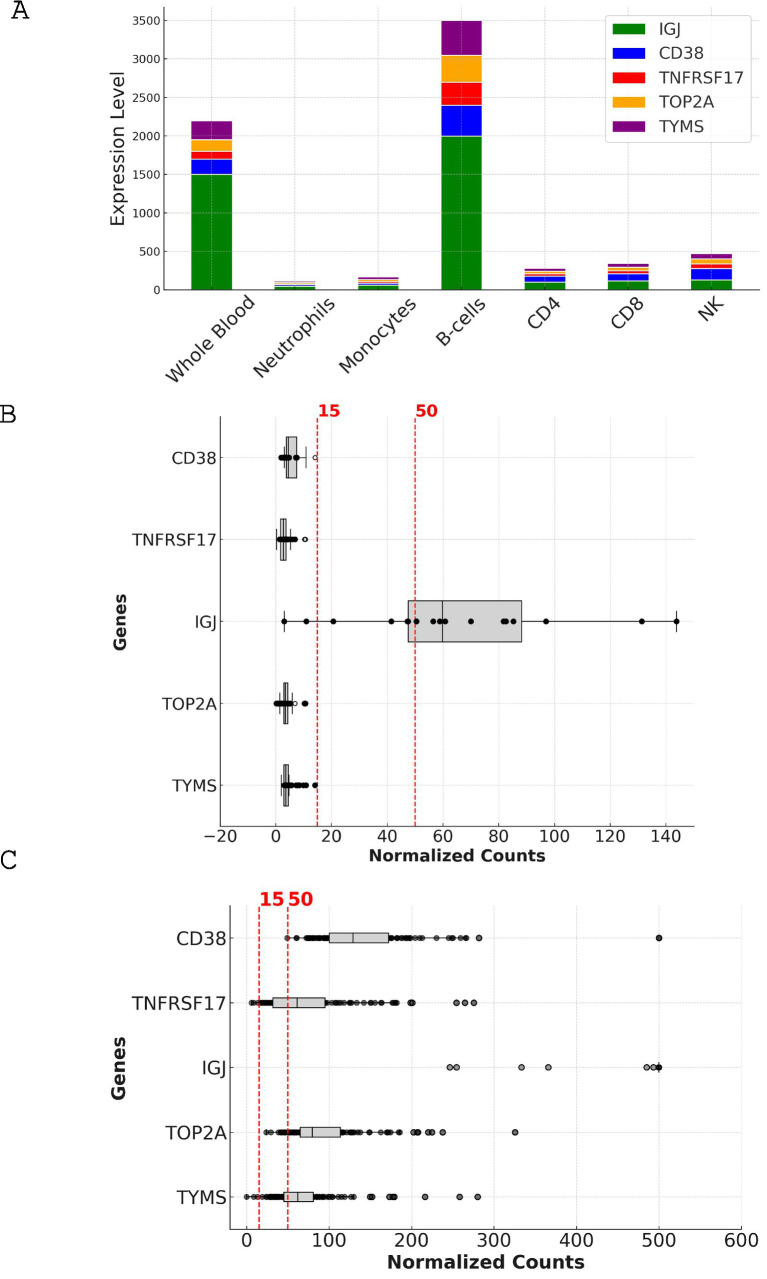
Expression analysis and comparison of top candidate genes in leukocyte-specific and MSP datasets. **(A)** Stacked bar chart depicting the expression levels of CD38, TNFRSF17, IGJ, TOP2A, and TYMS across different cell types in the leukocyte-specific dataset (GSE60424). The y-axis represents the expression level in normalized counts, while the x-axis shows the various cell types, including whole blood, neutrophils, monocytes, B cells, CD4, CD8, and NK cells. **(B)** Box plot illustrating the expression levels of the top candidate genes in whole blood samples from the leukocyte-specific dataset (GSE60424). The y-axis represents the expression level in normalized counts, while the x-axis shows the individual genes. The box plot displays the median, interquartile range, and outliers for each gene. The dotted lines indicate the 15-count and 50-count thresholds used for assessing the suitability of genes for reliable measurement in targeted assays. **(C)** Scatter plot depicting the expression levels of the top candidate genes across individual samples in the MSP dataset (PRJNA898879). The y-axis represents the expression level in normalized counts, while the x-axis shows the individual samples. The dotted line indicates the 15-count threshold used for assessing the suitability of genes for reliable measurement in targeted assays. The scatter plot highlights the variability in expression levels across samples and the higher overall expression of the candidate genes in the MSP dataset compared to the leukocyte-specific dataset.

To ensure the reliable measurement of selected genes in the targeted assay, we analyzed the expression levels of the top five candidate genes (CD38, TNFRSF17, IGJ, TOP2A, and TYMS) in two different datasets: a leukocyte-specific RNA-seq dataset (GSE60424) and an MSP dataset (PRJNA898879) (Figure 5 and Table 1).

**Table 1:** Flagging candidates with low expression (Step 5a).

Gene	Leukocytes (GSE60424)			MSP (PRJNA898879)		
	Median Expression	% of Samples > 15	Meets Criteria	Median Expression	% of Samples > 15	Meets Criteria
CD38	4.54	0	False	128.86	100	True
TNFRSF17	2.745	0	False	61.32	97.30	True
IGJ	59.8	0	True	1,549	100	True
TOP2A	3.5	0	False	79.71	100	True
TYMS	3.54	0	False	62.23	97.29	True

Figure 5A illustrates the expression levels of the candidate genes across various cell types and whole blood. IGJ, CD38, and TNFRSF17 show the highest expression in B-cells, followed by whole blood, consistent with their known roles in B-cell function and antibody production. In contrast, TOP2A and TYMS display lower expression across all cell types.

The box plot in Figure 5B represents the expression levels of the candidate genes in the leukocyte-specific dataset (GSE60424). IGJ demonstrates the highest median expression, followed by CD38 and TNFRSF17. TOP2A and TYMS show lower median expression levels. In this dataset, only IGJ met the criteria for reliable measurement, with a median expression of 59.8. The other genes, including CD38, TNFRSF17, TOP2A, and TYMS, showed lower median expression levels, ranging from 2.745 to 4.54, and did not meet the criteria (Table 1).

In contrast, the box plot in Figure 5C represents the expression levels of the candidate genes in the MSP dataset (PRJNA898879). All five genes show higher median expression levels compared to the leukocyte-specific dataset, with IGJ exhibiting the highest median expression at 1549, followed by CD38 at 128.86. TOP2A, TYMS, and TNFRSF17 also show high median expression levels (79.71, 62.23, and 61.32, respectively). In the MSP dataset, all five genes met the criteria for reliable measurement, with 100% of samples having expression levels above 15 for IGJ and CD38, and over 97% for TOP2A, TYMS, and TNFRSF17.

#### 3.4.2 Flagging candidates with low correlation to module average

**Figure 6:**
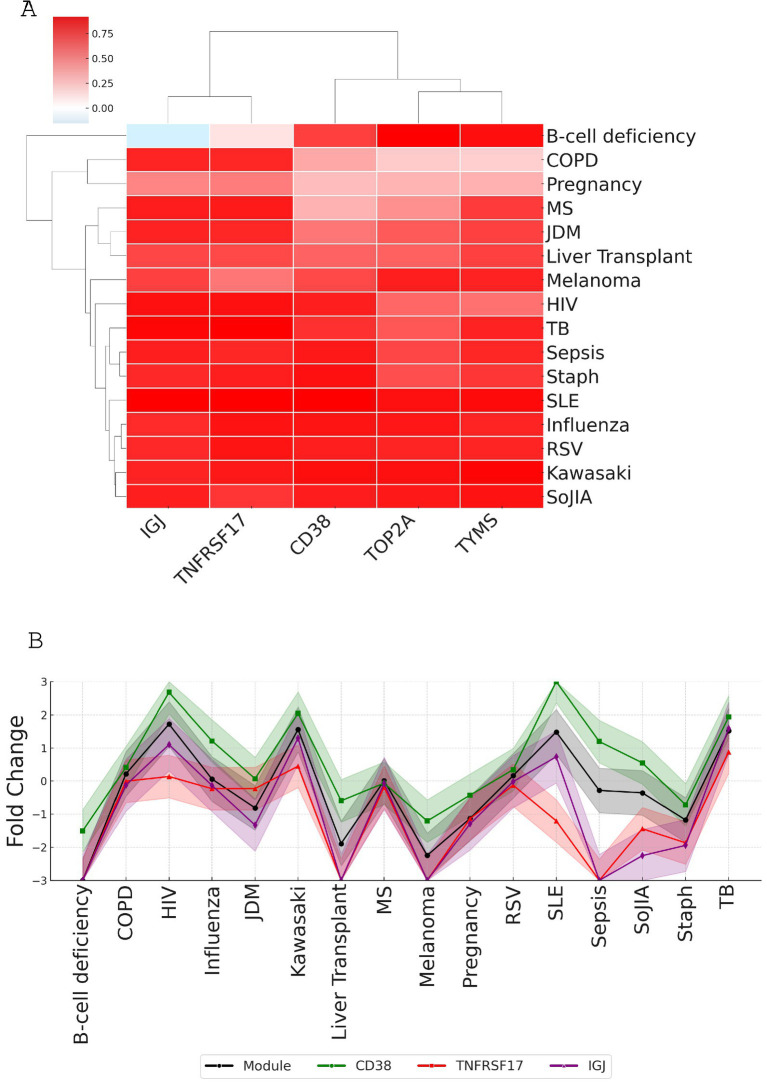
Correlation analysis of top candidate genes with module M12.15 average expression across various conditions. **(A)** Heatmap showing correlations between candidate genes (CD38, TNFRSF17, IGJ, TOP2A, and TYMS) and module M12.15 average expression across 16 medical conditions: patients with B-cell deficiency, chronic obstructive pulmonary disease (COPD), pregnancy, multiple sclerosis (MS), juvenile dermatomyositis (JDM), post-liver transplantation (liver transplant), melanoma, human immunodeficiency virus infection (HIV), tuberculosis (TB), sepsis, *Staphylococcus aureus* infection (Staph), systemic lupus erythematosus (SLE), influenza virus infection (influenza), respiratory syncytial virus infection (RSV), Kawasaki disease (Kawasaki), and systemic onset juvenile idiopathic arthritis (SoJIA). **(B)** Line plot comparing module average expression (black line) with individual candidate gene expression patterns across all conditions, demonstrating the strong correlation of CD38 with overall module behavior.

To select candidate genes representative of the entire module M12.15, we examined the correlation of each gene’s expression with the module average across different conditions using a microarray dataset covering 16 disease states and physiological conditions (GSE100150) (Figure 6A). The analysis shows that all five genes (IGJ, TNFRSF17, CD38, TOP2A, and TYMS) had strong correlations with the module average, with TOP2A having a slightly lower median correlation compared to the other genes (Table 2). When looking at each condition individually, the fold change of CD38 closely matches the expression of the module, further supporting its potential as a reliable representative of the module’s behavior in a wide range of physiological and disease states (Figure 6B).

**Table 2:** Flagging candidates with low correlation to module average (Step 5b).

Gene	Median correlation	Percentage above 0.5	Is low outlier	Meets both criteria
CD38	0.773	75	No	True
TNFRSF17	0.791	81.25	No	True
IGJ	0.789	87.5	No	True
TOP2A	0.649	81.25	No	True
TYMS	0.782	87.5	No	True

### 3.5 CD38 emerges as the top candidate gene from the M12.15 module

Through our comprehensive, multi-step prioritization process, CD38 consistently emerged as the top candidate gene from module M12.15, closely followed by TNFRSF17. Despite the relatively low expression levels of CD38 observed in the leukocyte-specific RNA-seq dataset (GSE60424), the MSP RNA-seq dataset (PRJNA898879) showed high expression levels for CD38, meeting the cut-off criteria for reliable measurement. Additionally, the microarray dataset (GSE100150) analysis revealed that CD38’s fold change closely matched the module’s expression pattern across various physiological and pathological conditions, supporting its potential as a reliable biomarker. The importance of CD38 in plasma cell biology and its potential as a therapeutic target warrant its inclusion in a targeted assay, even if its detection may require optimization of the assay conditions in certain contexts.

## 4 Discussion

In this study, we aimed to demonstrate the potential of LLMs in streamlining the knowledge-driven prioritization of candidate genes derived from systems-scale profiling data. We focused on module M12.15, a plasma cell-associated module from the BloodGen3 repertoire, which has been linked to various physiological and pathological conditions, including antibody responses to vaccines, autoimmune diseases, and certain hematological malignancies (14–17). By prioritizing the constituent genes of module M12.15 and selecting the most promising candidate for downstream characterization, we sought to showcase the utility of our automated LLM-based approach in enhancing the efficiency and scalability of candidate biomarker prioritization.

Our approach leveraged the capabilities of GPT-4, Claude 3.5, and Consensus GPT to score and rank candidate genes based on predefined criteria such as their association with plasma cell responses, relevance to leukocyte biology, potential as biomarkers, and therapeutic implications. Integrating this LLM-driven analysis with expression data from whole blood and leukocyte-specific datasets, we identified CD38, TNFRSF17, and IGJ as the top candidate genes, with CD38 emerging as a particularly promising target.

The value of our AI-human hybrid framework extends beyond merely confirming known plasma cell markers. While our analysis did identify CD38, TNFRSF17, and IGJ as top candidates, their selection emerged through a systematic, criterion-dependent evaluation process rather than predetermined expectations. Our detailed scoring analysis revealed the dynamic nature of genes marker rankings, which shifted based on specific evaluation criteria ranging from plasma cell biology to therapeutic relevance, demonstrating the capacity of the framework for nuanced, multi-dimensional assessment.

CD38, also known as cyclic ADP ribose hydrolase (22, 33), is a transmembrane glycoprotein involved in various biological processes, including cell adhesion, signal transduction, and calcium signaling (34–36). It is strongly associated with plasma cell responses, being highly expressed on plasma cells and involved in their survival and proliferation (34, 37). CD38 has established clinical relevance as a biomarker (38–45) and therapeutic target, particularly in multiple myeloma, where anti-CD38 antibodies like daratumumab have shown significant efficacy (38). The biological significance and clinical relevance of CD38 in plasma cell-related disorders (40–43), along with its consistent high scores across our prioritization process, make it a compelling candidate for inclusion in a targeted assay.

The role of CD38 in multiple myeloma extends beyond its utility as a plasma cell marker. Recent studies have revealed its complex functions in the bone marrow microenvironment (46) and its impact on disease progression (47). The clinical efficacy of anti-CD38 monoclonal antibodies, such as daratumumab and isatuximab, has been demonstrated in various phases of multiple myeloma treatment, including newly diagnosed, relapsed, and refractory settings (47–53). These antibodies function through multiple mechanisms, including complement-dependent cytotoxicity (CDC), antibody-dependent cellular cytotoxicity (ADCC), and direct induction of apoptosis (54–57). In addition, anti-CD38 therapy has been shown to deplete CD38-expressing immunosuppressive cells, further enhancing antitumor immune responses (48, 58, 59). Given the multifaceted biological roles of CD38 and its therapeutic implications, its inclusion in targeted assays can facilitate a more comprehensive understanding of plasma cell-related disorders and improve the precision of therapeutic interventions.

In our analysis, we used a cut-off of 15 read counts and a median of 50 read counts to determine the suitability of genes for reliable measurement in targeted assays. The 15 read count cut-off was chosen based on earlier studies suggesting that expression levels below 10 counts are considered background or very low (60). In our previous study (MSP), we arbitrarily selected a median of 50 read counts as a cut-off to filter out low-expressed genes (4) when constructing a panel of 192 genes. After panel construction, we observed that all genes with a median expression above this threshold were consistently detectable using high-throughput RT-PCR (data not shown). These thresholds ensure that the selected genes have sufficient expression levels to be reliably measured in targeted assays, reducing the risk of false negatives and ensuring the reproducibility of results.

Despite its promising performance in the LLM-based prioritization, our analysis of expression data revealed that CD38 has relatively low expression levels in the leukocyte-specific dataset (GSE60424), failing to meet the cut-off criteria for reliable measurement. This finding highlights the potential challenges in detecting CD38 in blood-based assays using this dataset alone. However, in contrast, the MSP RNA-seq dataset (PRJNA898879) demonstrated high expression levels for CD38, meeting the cut-off criteria and suggesting its suitability for reliable measurement in the context of pregnancy. These findings highlight the importance of integrating data-driven approaches with knowledge-based prioritization to ensure the technical feasibility of detecting candidate genes in targeted assays. While sensitive methods like RT-PCR or RNA-seq with high sequencing depth might still allow for the reliable detection of CD38, its low expression levels in certain contexts warrant careful validation before final selection.

In the event that CD38 proves to be challenging to detect reliably, TNFRSF17 and IGJ, which also showed strong performance in our prioritization process, could serve as potential alternative candidate genes for module M12.15. TNFRSF17, also known as B-cell maturation antigen (BCMA), plays a critical role in the survival and differentiation of plasma cells and has emerged as a promising therapeutic target for multiple myeloma and other plasma cell disorders (61–74). IGJ, on the other hand, exhibited higher expression levels and strong correlations with the module average across various conditions in our analysis. As a key component of the secretory immunoglobulin complexes IgM and IgA, IGJ plays a crucial role in the assembly and transport of these antibodies, which are essential for the functions of plasma cells (75–78).

Our current study builds upon and significantly enhances the workflow from our previous publications (13, 79) by introducing a novel two-step process that combines AI-driven analysis with human expertise. This approach not only incorporates additional data-driven steps but also introduces human-augmented scoring and generation, addressing key limitations of relying solely on LLM-based knowledge synthesis. The first step involves an initial high-throughput screening to identify top-tier candidate genes using multiple LLM approaches. The second step employs high-resolution scoring and concurrent fact-checking. In this step, human experts actively validate and refine AI-generated scores, ensuring accuracy and relevance. This human-in-the-loop process allows for real-time adjustments based on expert knowledge, significantly enhancing the reliability of our gene prioritization.

Furthermore, we have incorporated additional data-driven steps to provide a more comprehensive evaluation of gene suitability for targeted assays. Step 5a, which focuses on evaluating expression levels in whole blood, addresses the critical issue of technical feasibility by ensuring that selected genes have sufficient expression for reliable measurement in targeted assays. Step 5b, which assesses the correlation of each gene with the module average across whole blood transcriptome datasets, ensures that selected genes consistently represent the module’s behavior across various physiological and pathological states.

Importantly, our integrated approach demonstrated the value of balancing statistical significance with biological relevance and clinical utility. For instance, while purely statistical analysis of expression data might prioritize genes like TOP2A and TYMS based on strong fold changes or correlation coefficients, our framework revealed their limited biological association with plasma cell function. This highlights the importance of considering multiple dimensions when selecting candidates for targeted assays, ensuring that the chosen genes are not only statistically significant but also biologically relevant to the context of interest.

In conclusion, our study demonstrates the successful development of an AI-human hybrid framework for systematic gene prioritization, with implications extending beyond the identification of plasma cell markers. The significance of our findings lies in establishing a structured methodology that combines multiple analytical approaches, providing detailed, criterion-specific assessments through multiple analytical approaches. This systematic process ensures consistent evaluation while maintaining the flexibility to address various research contexts and priorities, validating its potential for analyzing diverse modules across the BloodGen3 repertoire.

Despite our study demonstrated the utility of LLMs in candidate gene prioritization and selection, it is important to acknowledge its limitations. The performance of LLMs is dependent on the quality and scope of their training data, and they may not capture the most recent findings or niche areas of research. Additionally, the LLM-generated information is not always factual, requiring manual curation and fact-checking. Furthermore, the relative importance of the criteria used for gene prioritization may vary depending on the specific research question or clinical application, which might require the adjustment of weights.

Future research should focus on further validating and refining the AI-human hybrid framework across a broader range of biological contexts and module repertoires. This could involve applying the framework to less well-characterized modules, assessing its performance in identifying novel biomarker candidates, and comparing its results with those obtained through traditional data-driven approaches. Additionally, exploring the integration of more advanced AI techniques, such as few-shot learning or transfer learning, could further enhance the adaptability and efficiency of framework in handling diverse datasets and research questions.

## Data Availability

The datasets analyzed during the current study are available in the Gene Expression Omnibus (GEO) repository (GSE60424 and GSE100150) and Sequence Read Archive (PRJNA898879).

## Footnotes

^1^ https://chatgpt.com/g/g-bo0FiWLY7-consensus
